# The long non-coding RNA H19 - a new player in hepatocellular carcinoma

**DOI:** 10.15698/cst2017.10.102

**Published:** 2017-10-01

**Authors:** Maximilian A. Ardelt, Johanna Pachmayr

**Affiliations:** 1 Department of Pharmacy, Pharmaceutical Biology, University of Munich, Munich, Germany.; 2 Pharmacy, Pharmaceutical Biology & Clinical Pharmacy, Paracelsus Medical University, Salzburg, Austria.

**Keywords:** lncRNA, H19, HCC

Hepatocellular carcinoma (HCC) is among the most prevalent and lethal cancers worldwide 1. Incidence is increasing and due to its high chemoresistance, the therapeutic options are limited and HCC has risen to become one of the leading causes of cancer-related death 2. Thus, a detailed characterization of the mechanisms of HCC chemoresistance is urgently required in order to find new therapeutic approaches to overcome HCC chemoresistance.

The study by Schultheiss *et al*. suggests an interesting player to overcome chemoresistance in HCC: the long non-coding RNA (lncRNA) H19 3. LncRNAs have recently emerged as pivotal players in cancer, due to their contribution to cancer development, metastasis and chemoresistance 4. H19 was associated with various types of cancer, but its role in cancer is conflicting, as tumor-promoting and tumor-suppressive actions have been demonstrated 5,6,7. H19 was shown to be regulated under inflammatory conditions 5 and has been linked with HCC that represents a type of tumor that is associated with inflammatory conditions such as found in viral hepatitis as well as in alcoholic and non-alcoholic steatohepatitis 8,9,10. However, the reports about H19 in HCC are contradictory and H19 was described both to promote as well as to suppress tumor development 9,10,11. Thus, the role of H19 in HCC is still unclear.

In order to investigate the expression of H19 in HCC, Schultheiss *et al*. used four independent patient cohorts which revealed a decreased expression of H19 in human HCC tissue compared to non-tumorous tissue. However, interestingly, although H19 was downregulated in HCC, in each of the investigated patient cohorts a high H19 expression was observed in a small patient subgroup. This might explain the contradictory findings about H19 expression in small patient cohorts. In line with H19 levels, the expression of miR 675, which is encoded by H19 12,13, was decreased, whereas the expression of the mRNA binding protein EVAL1, a negative regulator of miR-675, was increased in HCC tissues.

Subsequently, the authors addressed the mechanisms underlying the decreased expression of H19 in HCC. H19 expression can be regulated by loss of imprinting (LOI) and by differential promoter methylation 14. Schultheiss *et al*. showed that the decreased H19 expression was not due to LOI. Interestingly, H19 promoter methylation was decreased in HCC compared to normal liver tissue, suggesting that decreased expression of H19 correlates with decreased promotor methylation. However, elevated gene expression is normally based on decreased promoter methylation. Nevertheless, an association of hypermethylation and increased gene expression has been described as well 15. Thus, the relationship between H19 promotor methylation and expression in HCC needs further investigation and will be an interesting topic for further research.

To further determine the function and regulation of H19 in HCC, the authors used three different HCC cell lines, including HepG2, Plc/Prf/5, and Huh7. H19 overexpression impaired clonogenic growth and improved the response of HCC cells to the treatment with sorafenib and doxorubicin, suggesting a chemosensitizing effect of H19. However, determination of the influence of H19 overexpression and knockdown on cell viability and proliferation revealed cell line specific differences: except for an elevation of viability of HepG2 cells, HCC cell viability was not affected by H19 overexpression or knockdown. Moreover, whereas proliferation of HepG2 cells was not affected, H19 overexpression reduced proliferation of Plc/Prf/5 and Huh7 cells. These data suggest a rather context-dependent function of H19 in HCC. Investigating H19 signaling suggested that neither miR-675 nor ELAV1 or IGF2 contributed to the sensitizing effect of H19, as these components were not modulated in cells with H19 upregulation. Thus, the signaling network of H19 in HCC remains an interesting question for future research.

In order to evaluate whether H19 contributes to chemoresistance, Schultheiss *et al*. generated doxorubicin- and sorafenib-resistant HepG2, Plc/Prf/5 and Huh7 cells. In fact, downregulation of H19 in the resistant cell lines suggested an association of H19 suppression with chemoresistance. Moreover, H19 promotor methylation was changed during chemoresistance, but again cell-line specific differences were observed: all three sorafenib-resistant cell lines as well as doxorubicin-resistant HUH7 cells showed elevated promoter methylation with differences in the specific CpG sites, whereas doxorubicin-resistant HepG2 and Plc/Prf/5 cells showed a heterogenous hyper- or hypomethylation of the different CpG sites. As most of the changes were observed nearest to the transcription start site, the authors suggested that the modified promotor methylation contributes to decreased H19 expression during chemoresistance. Therefore, Schultheiss *et al*. investigated the influence of the DNA-demethylating agent 5-azacytidine on HCC cell chemoresistance. 5-azacytidine decreased H19 promoter methylation and increased H19 expression in HepG2 and Huh7 cells but not in Plc/Prf/5 cells. Moreover, 5-azacytidine sensitized HCC cells to doxorubicin treatment. As H19 overexpression improved cell death induction by doxorubicin and inhibited proliferation of chemoresistant HCC cells, H19 overexpression was suggested as potential approach to overcome chemoresistance in HCC.

Finally, the influence of H19 on tumor induction was investigated. In fact, H19 deficient mice demonstrated increased tumor development by the carcinogen diethylnitrosamine (DEN) with tumors that showed increased cell proliferation as well as dysplastic lesions characteristic for liver carcinogenesis.

In summary, the study by Schultheiss *et al*. shows that the lncRNA H19 is downregulated in HCC tissues and that it suppresses chemoresistance and tumorigenesis. The contribution of H19 promotor methylation and the cell-line specific effects of H19 open exciting questions for future research. To conclude, the study by Schultheiss *et al*. helps to understand the function of H19 in HCC and suggests H19 as a potential target to overcome chemoresistance in HCC.
